# Comparison of Visuospatial and Verbal Abilities in First Psychotic Episode of Schizophrenia Spectrum Disorder: Impact on Global Functioning and Quality of Life

**DOI:** 10.3389/fnbeh.2015.00322

**Published:** 2015-12-18

**Authors:** Mabel Rodriguez, Filip Spaniel, Lucie Konradova, Katerina Sedlakova, Karolina Dvorska, Jitka Prajsova, Zuzana Kratochvilova, David Levcik, Kamil Vlcek, Iveta Fajnerova

**Affiliations:** ^1^National IT System of Mental Health and Brain Monitoring, National Institute of Mental HealthKlecany, Czech Republic; ^2^Department of Psychiatry and Medical Psychology, Third Faculty of Medicine, Charles University in PraguePrague, Czech Republic; ^3^Department of Neurophysiology of Memory, Institute of Physiology of the Czech Academy of SciencesPrague, Czech Republic

**Keywords:** cognitive deficit, first psychotic episode, schizophrenia spectrum disorder, global functioning, quality of life, visuospatial functions, verbal functions, antipsychotic medication

## Abstract

**Objectives:** Deficit in visuospatial functions can influence both simple and complex daily life activities. Despite the fact that visuospatial deficit was reported in schizophrenia, research on visuospatial functions as an independent entity is limited. Our study aims to elucidate the impact of visuospatial deficit in comparison with verbal deficit on global functioning and quality of life in the first psychotic episode of schizophrenia spectrum disorder (FES). The significance of clinical symptoms and antipsychotic medication was also studied.

**Methods:** Thirty-six FES patients and a matched group of healthy controls (HC group) were assessed with a neuropsychological battery focused on visuospatial (VIS) and verbal (VERB) functions. Using multiple regression analysis, we evaluated the cumulative effect of VERB and VIS functions, psychiatric symptoms (PANSS) and antipsychotic medication on global functioning (GAF) and quality of life (WHOQOL-BREF) in the FES group.

**Results:** The FES group demonstrated significant impairment both in VIS and VERB cognitive abilities compared to the HC group. Antipsychotic medication did not significantly affect either VIS or VERB functioning. PANSS was not related to cognitive functioning, apart from the Trail Making Test B. In the FES group, the GAF score was significantly affected by the severity of positive symptoms and VERB functioning, explaining together 60% of GAF variability. The severity of negative and positive symptoms affected only the Physical health domain of WHOQOL-BREF. The degree of VERB deficit was associated with both Physical and Psychological health. Although we did not find any relation between VIS functioning, GAF, and WHOQOL-BREF, a paradoxical finding emerged in the Environment quality domain, where a worse quality of the environment was associated with better VIS functioning.

**Conclusions:** Our results suggest that the deficit in VIS functions is an integral part of cognitive deficit in schizophrenia spectrum disorders, rather than a side effect of symptomatology or antipsychotic medication. Moreover, VERB functioning was a better predictor of GAF and WHOQOL-BREF than VIS functioning. Given the findings of negative or missing effect of VIS deficit on WHOQOL-BREF and GAF, the accuracy of these measures in evaluating the impact of global cognitive deficit on everyday life in schizophrenia could be questioned.

## Introduction

Abnormalities in cognitive functions are considered to be one of the key components in Schizophrenia (SZ). Neurocognitive deficit represents a reliable feature, with moderate to large effect size on global functioning across all cognitive domains (Milev et al., 2005). An important amount of research in SZ has examined the relationship between cognition and variety of clinical factors including age of onset, symptomatology, severity, duration, medication, functional outcome, and Quality of life (QOL) (Heinrichs and Zakzanis, 1998; Bilder et al., 2000; Mesholam-Gately et al., 2009). However, studies focusing on specific relationships among cognitive domains or relationships inside the domains and their influence on daily functioning are limited (Harvey et al., 2012). One of the most neglected areas is the comparison of the impact that visuospatial (VIS) and verbal (VERB) abilities have on global functioning and QOL. Focused research of VIS functions can help provide a better understanding of neuropsychological patterns of heterogeneity in SZ. In addition, since VIS functions are less biased by language skills, research in this area can enlarge the neuropsychiatric field (Paradis, 2008). Furthermore, VIS functions are an important tool for a comparative research on animal models of SZ (Fajnerová et al., 2014).

One of the main reasons why visuospatial functions did not receive much attention as an independent entity in the research of SZ was the negative impact of the first-generation (typical) antipsychotic treatment on some motor and visuospatial functions (e.g., psychomotor retardation; Spohn and Strauss, 1989; Bilder et al., 1992; Meltzer and McGurk, 1999; Arana, 2000). With the development of second generation (atypical) antipsychotics (AP), the risk of neurological side effects seemed reduced. Moreover, findings of slightly positive effects of some atypical AP on cognitive deficit were reported in SZ (e.g., Peuskens et al., 2005; Houthoofd et al., 2008) and these findings were supported by results in an animal model of SZ (Bubeníková et al., 2005). However, when atypical antipsychotics were directly compared with the typical ones, no differences were found in either psychomotor functions or other cognitive areas (Jones et al., 2006; Keefe et al., 2007; Lewis and Lieberman, 2008). Another limitation related to the study of VIS functions is that not all studies use a separate model of verbal and visuospatial functions. Usually, both VERB and VIS functions are included in the same cognitive domain (e.g., memory domain) or in the total IQ score; alternatively, they are studied as isolated variables. The question how much (if at all) the AP medication affects the motor and VIS functions when compared with the verbal functions, and to what extent the visuospatial functions impact daily functioning and the quality of life requires more research.

Visuospatial impairment can negatively affect various daily activities from the most common, such as watching TV or reading a book, to the most complex, including social interactions (visual recognition of social signals), and recognition of territorial boundaries (interpersonal space; Cummings and Mega, 2003). SZ subjects exhibit impaired performance in a wide range of VIS functions, from the most basic level of visual perception to more complex visuospatial processing and navigation abilities (e.g., Stuve et al., 1997; Doniger et al., 2001; Butler et al., 2005; Hanlon et al., 2006; Piskulic et al., 2007; Weniger and Irle, 2008; Cocchi et al., 2009; Folley et al., 2010; Landgraf et al., 2010; Fajnerová et al., 2014). This decline in VIS performance is already present in the first episode of schizophrenia and performance further deteriorates over time, predicting poor outcome (Stirling et al., 2003). Cross-sectional studies in subjects with late-life schizophrenia report the impairment in visuospatial ability, alongside with the executive and verbal fluency deficit. Moreover, longitudinal studies suggest that the cognitive decline in late-life schizophrenia may first affect VIS abilities (Rajji and Mulsant, 2008). It was also demonstrated that VIS tasks related to attention, memory, and planning predict improvements on psychosocial functions, such as autonomy in daily living, treatment compliance, and social competence in subjects with psychosis (Prouteau et al., 2005). Given the significance of VIS functions in our daily life, it is expected that visuospatial tests would be good predictors of functional outcome in SZ.

Functional capacity and quality of life play a key role in the study of the course, treatment efficacy, and other factors related to functional outcome in SZ. Both functional capacity and QOL are negatively associated with clinical symptoms (Gaite et al., 2005; Malla and Payne, 2005; Milev et al., 2005; Makara-Studziñska et al., 2011). Negative symptoms are more strongly related to poor QOL and psychosocial functioning in SZ outpatients (Eack and Newhill, 2007; Rocca et al., 2009), whereas general psychopathology shows a consistent negative relationship with QOL across all study samples and treatment settings (Eack and Newhill, 2007; Rocca et al., 2009). Findings about the influence of positive symptoms are heterogenous, with the relationship toward negative and general symptomatology being more evident, varying only in the extent of impact (Eack and Newhill, 2007; Rocca et al., 2009).

In terms of cognition, the current state of the literature did not enable drawing any conclusions about specific cognitive constructs related to global functioning and QOL. Some studies have documented a relationship between measured general cognitive ability (IQ index) and global functioning or QOL (Tzeng et al., 2004; Chaplin et al., 2006; Leeson et al., 2009), while other studies also point out the importance of specific neuropsychological domains. Results of these studies are very heterogeneous, depending on the measures used in the assessment. Despite the heterogeneity of measures, the deficit in executive functions appears to be the most evident and burdensome, and is most related to the impairment of global functioning and QOL in SZ (Bilder et al., 2000; Reed et al., 2002). In addition, lower QOL is related to the deficit in verbal memory (Ritsner, 2007; Fiszdon et al., 2008; Matsui et al., 2008). As was stated previously, VIS and VERB functions are usually combined in a single domain or IQ score. Thus, the role of VIS functions in global functioning and QOL remains unclear.

To our knowledge, no study to date has described the extent to which visuospatial functions affect everyday life of SZ patients, in contrast to the effect of verbal abilities. Our study aimed to answer the following questions:

(1) Are visuospatial abilities impaired in the first-episode schizophrenia spectrum (FES) patients in comparison with the matched group of healthy controls? If that is the case, is the degree of the deficit the same as in verbal functions?
(2) Are the VIS functions in FES patients affected by the antipsychotic medication and the actual psychiatric symptomatology (measured with PANSS)? Is similar effect visible in the VERB functions?
(3) Is the global functioning and the quality of life in FES patients affected by VIS functioning when analyzed in the presence of VERB functions and clinical characteristics (symptoms and medication dose)? If so, is the effect of visuospatial and verbal functioning the same?

## Materials and methods

### Subjects

Thirty-six subjects (22 males and 14 females, FES group) who met ICD-10 criteria for the first psychotic episode of schizophrenia spectrum disorder [F20.X (*N* = 4) and F23.1/F23.2 (*N* = 32)] were recruited at the National Institute of Mental Health (NIMH). Patients were evaluated once they were stabilized at the end of their first psychiatric hospitalization in partial symptomatic remission state, according to Andreasen's remission criteria (2005). The group was considered in partial remission state rather than in complete remission, as they did not fulfill the criterion of asymptomatic 6-month period. Study subjects were diagnosed in a routine clinical process by two experienced psychiatrists. In case of diagnostic disagreements (e.g., comorbidity) the specific case was excluded from the study.

In order to compare the cognitive performance in FES subjects with the healthy population, a group of healthy control subjects (*N* = 36, group HC) was recruited from the same socio-demographic background via a local advertisement. The inclusion criteria for both groups were: (a) 17–35 years of age; (b) no history of neurological disease or loss of consciousness longer than 10 min; (c) native in Czech/Slovak language; and (d) additionally for the FES group to meet ICD-10 diagnostic criteria (dg F20.X or F23.1, F23.2) and to be first admitted to psychiatric care. The main exclusion criterion for the control subjects was personal history of any psychiatric disorder; for the FES group it was the fulfilled diagnostic criterions for another psychiatric disorder. Both groups were carefully matched in terms of sex, age (max 2 years difference tolerance), and level of education (for details and statistical comparison of the matching parameters, see Table 1). In each group there were 16 participants with a higher education level (university studies) and 20 with a lower level of education.

**Table 1:** **Demographic data, clinical assessment, and QOL questionnaire**.

**Demographic variables**	**Group mean** ± **SD**		**Group differences**	
	**FES**	**HC**	**Mann–Whitney *U***	***p*-value**
N	36	36		
Sex (M:F)	22:14	22:14		
Age	26.3 ± 5.6	25.7 ± 5.2	614	0.697
Education level (1–6)	3.7 ± 1.3	4.0 ± 1.2	556	0.261
**Clinical assessment**	**FES subjects (mean ± SD)**			
PANSS total score	50.8 ± 17			
PANSS-positive	12.5 ± 5.2			
PANSS-negative	16.0 ± 7.3			
PANSS-general	26.4 ± 6			
AP medication—CPZ equivalents (mg)	391.2 ± 122			
GAF	64.5 ± 18.3			
**WHOQOL-BREF**	**FES subjects (mean ± SD)**		**Normative data (mean ± SD) (Dragomirecká and Bartoňová, 2006b)**	
Physical health (domain 1)	14.4 ± 2.4		15.5 ± 2.6	
Psychological health (domain 2)	14.1 ± 2.5		14.8 ± 2.4	
Social (domain 3)	13.2 ± 3.1		15.0 ± 2.9	
Environmental (domain 4)	14.3 ± 2.1		13.3 ± 2.1	

*First psychotic episodes of schizophrenia spectrum disorder subjects (FES) and healthy controls (HC) individually matched by sex, age (within 2 years), and education level (see demographic variables). All values are present as mean ± SD. Clinical scales PANSS (Positive and Negative Symptoms Scale) and GAF (Global Assessment of Functioning), antipsychotic medication level in CPZ (chlorpromazine) equivalents, and Quality of life questionnaire WHOQOL-BREF assessed in FES subjects. Education level: 1, less than high school; 2, started high school; 3, completed high school; 4, started university; 5, completed university; 6, started postgraduate studies*.

Prior to the study, all participants signed a written informed consent in accordance with the Declaration of Helsinki, approved by the Ethics Committee of NIMH.

### Clinical and neuropsychological assessment

Two psychiatric scales were used to evaluate clinical characteristics in the FES subjects. Current symptomatology was assessed using the Positive and Negative Syndrome Scale (PANSS; Kay et al., 1987). The Global Assessment of Functioning (GAF; Jones et al., 1995) was used in order to objectively evaluate general psychosocial functioning of the FES group. The GAF scale is used to address general functioning (score 0–100) in daily activities of individual FES subjects. All FES subjects were medicated by different dose and type of atypical antipsychotics or their combination (olanzapin, amisulpirid, and risperidon), that is why chlorpromazine equivalents (CPZ; Woods, 2003; Andreasen et al., 2010) were used to evaluate the effect of medication dosage on cognitive functioning. For details on the clinical parameters see Table 1.

The quality of life was subjectively evaluated by FES subjects using the Quality of life questionnaire WHOQOL-BREF (WHO group, 1998), a short-form quality of life assessment that calculates four domain profiles (Physical health, Psychological health, Social relationships, and Environment), and was validated for FES population (Mas-Expósito et al., 2011). The questionnaire was translated and validated for a Czech population (Dragomirecká and Bartoňová, 2006b).

Regarding the neuropsychological assessment, the used measures were chosen in accordance with the evidence of related articles (mentioned in introduction) and suggested also by the MATRICS initiative (Green et al., 2004; Nuechterlein et al., 2008). Some additional measures not commonly used and standardized in schizophrenia population were used in order to assess VIS functions in greater detail. The final neuropsychological battery consisted of 11 tests focused on both the visuospatial and the verbal functions (see Table 2). All tests were assessed by trained clinicians, according to the cited administration protocols. Detailed information about all test methods is provided in Table 2. In order to compare cognitive performance in the FES subjects with the healthy population, the same test battery was administered to a HC subjects. Below is a description of the three visuospatial methods that have some specific characteristics.

**Table 2:** **Description of (A) Visuospatial and (B) Verbal neuropsychological tests**.

**Test**	**Monitored cognitive function**	**Test outputs**	**References**	**Test description**
**(A) VISUOSPATIAL NEUROPSYCHOLOGICAL TESTS**				
Trail Making Test (TMT A and B)	Psychomotor speed (A); visuospatial working memory (B); mental flexibility (B/A)	Time A (s); Time B (s); Ratio B/A	Reitan and Wolfson, 1985; Preiss and Preiss, 2006	Chaining a sequence of numbers (A) or alternatively numbers and letters (B) that are randomly distributed on a single paper
Rey-Osterrieth (Taylor) Complex Figure Test (RCFT)	Visuospatial organization, constructional functions and visual memory	Raw score for copy trial (RCFT-copy), reproduction after 3 (RCFT-3) and 30 min (RCFT-30)	Osterrieth, 1944; Preiss et al., 2012	Copy and reconstruction of figure after 3 and 30 min
Key Search Test (KST)	Executive functions	Raw scores of strategy	BADS (Wilson et al., 1996)	Strategy of exploration of 2-dimensional space (2D square shape)
Money Road-Map Test (RMT)	Spatial orientation	Raw scores for number or errors/32; A, B and C error types	Money et al., 1965	Ability to determine right/left turns on crossroads in 2D view of a simple maze/city
Spatial Span (SS)	Visuospatial working memory	Raw scores: total (forward + backward)	PC version adjusted from the Corsi block test in (PEBL, 2012) according to WMS-III (Wechsler, 1997)	Repeating a sequence of spatial positions presented in 2D plane, forward or backward
PEBL Perceptual Vigilance Task (PVT)	Vigilance and attention	Number of lapses [Reaction time (RT) over 500 ms], average RT speed	PEBL battery (PEBL, 2012; Dinges et al., 1985; Loh et al., 2004)	Response to stimulus appearing in the variable time interval (1–9 s) during 10 min
**(B) VERBAL NEUROPSYCHOLOGICAL TESTS**				
Auditory Verbal Learning Test (AVLT)	Verbal learning and memory	Learning curve and total number of words (AVLT-I-V); immediate recall (AVLT-VI); delayed recall (AVLT-30); number of confabulations and repetitions	Rey, 1964; Preiss et al., 2012	Repeated recall of 15 words with interference trial (B) and delayed recall after 30 min
Verbal Fluency Test (VFT)	Psychomotor speed and mental flexibility	Number of words for phonemic (total of three trials) and semantic fluency	Preiss et al., 2012	Speaking aloud words beginning with letters N, K, P or naming category of animals during 1 min
Digit Span (DS)	Attention (forward), verbal working memory (backward)	Raw scores: total (forward + backward)	WAIS-III (Wechsler, 1997); Czech version (Černochová et al., 2010)	Repeating list of numbers forward and backward
Similarities (Sim)	Verbal conceptualization	Raw score–correct responses		Describe similarities between pair of words

The PEBL (PEBL, 2012) version of the Perceptual Vigilance Task (PVT) was used in its 10-min-long alternative (Loh et al., 2004) in order to test attention and vigilance. A simple circle stimulus appears in the PVT at intervals ranging between 2 and 12 s, and the participant is required to press the spacebar as quickly as possible.

A computerized version of the Spatial Span (SS) test was used in order to test spatial attention and working memory without uncontrolled examiner effects (such as prolonged presentation of the longer spatial sequences). We adjusted the original protocol of the Corsi block-tapping test (Kessels et al., 2000) applied in the PEBL battery (PEBL, 2012) to match individual positions and spatial sequences of the SS in the WMS-III (Wechsler, 1997; Černochová et al., 2010).

The Money Road-Map-Test (Money et al., 1965) is not traditionally used in standard test batteries such as MATRICS; this test was selected in order to test specific visuospatial functions, such as mental rotation and perspective taking strategy. For this reason, the total number of errors (out of a total of 32 turns) was divided into three categories (according to Marková et al., 2015) by the angle of the route before each turn relative to the subject's heading: (A) rotation of < 70° (9 turns), (B) rotation of 90° (13 turns), and (C) rotation of more than 110° (10 turns). Another specifically selected test, the Key Search Test, was chosen as a sensitive method testing dysexecutive syndrome in schizophrenia (Evans et al., 1997), using spatial planning abilities.

### Data analysis

Statistical analysis was performed using the SPSS software (version 15.0). The significance level of all statistical analysis was set to 0.05. The group differences in demographic variables (age, education) were analyzed using non-parametric Mann–Whitney *U*-test. Identical method was used to compare the raw scores obtained in the visuospatial tests. Non-parametric Spearman Rank Order Correlations were used in order to detect correlations between variables.

The raw scores of neurocognitive tests were used to compare performance between the FES subjects and the HC. Raw scores were transformed to z-scores in order to calculate cumulative scores of VERB and VIS scores. Z-scores were calculated as the difference among raw scores of the individual FES subjects and the HC group mean, divided by the HC standard deviation. The cumulative scores (VERB and VIS) were computed as a sum of the standardized z-scores divided by the number of applied measures from the relevant variables list as follows (for explanation of individual abbreviations see Table 2): VERB score (AVLT_I-V, AVLT-VI, AVLT-30, VFT-semantic, VFT-phonemic, DS-backward, Similarities) and VIS score (RCFT-copy, RCFT-3, RCFT-30, TMT-A, TMT-B, SS-backward, RMT-total errors). PVT test results were not included in calculation of the cumulative scores, as performance on this test is purely attentional. In addition, to assure the accuracy of input variables to the cumulative scores and their consistency, reliability analysis and factor analysis were performed (Cronbach's Alpha for VERB = 0.73; Cronbach's Alpha for VIS = 0.75). Multiple linear regression analysis (stepwise method criteria as follows: probability-of-F-to-enter ≤ 0.05; probability-of-F-to-remove ≥ 0.10) was used to assess the effect of performance in individual visuospatial and verbal tests (dependent variables) of (A) the clinical characteristics (independent variables): PANSS (scores divided into three sub-scores: general symptoms–G, positive symptoms–P, negative symptoms–N), and (B) the antipsychotic medication calculated in CPZ. In additional stepwise multiple linear regression analysis an overall effect of PANSS scores, CPZ level, and cumulative VERB and VIS scores was assessed on (A) global functioning measured by GAF and (B) four individual domains of quality of life measured by WHOQOL-BREF (WHO group, 1998).

## Results

### Differences in cognitive performance between FES and HC group

**Figure 1:**
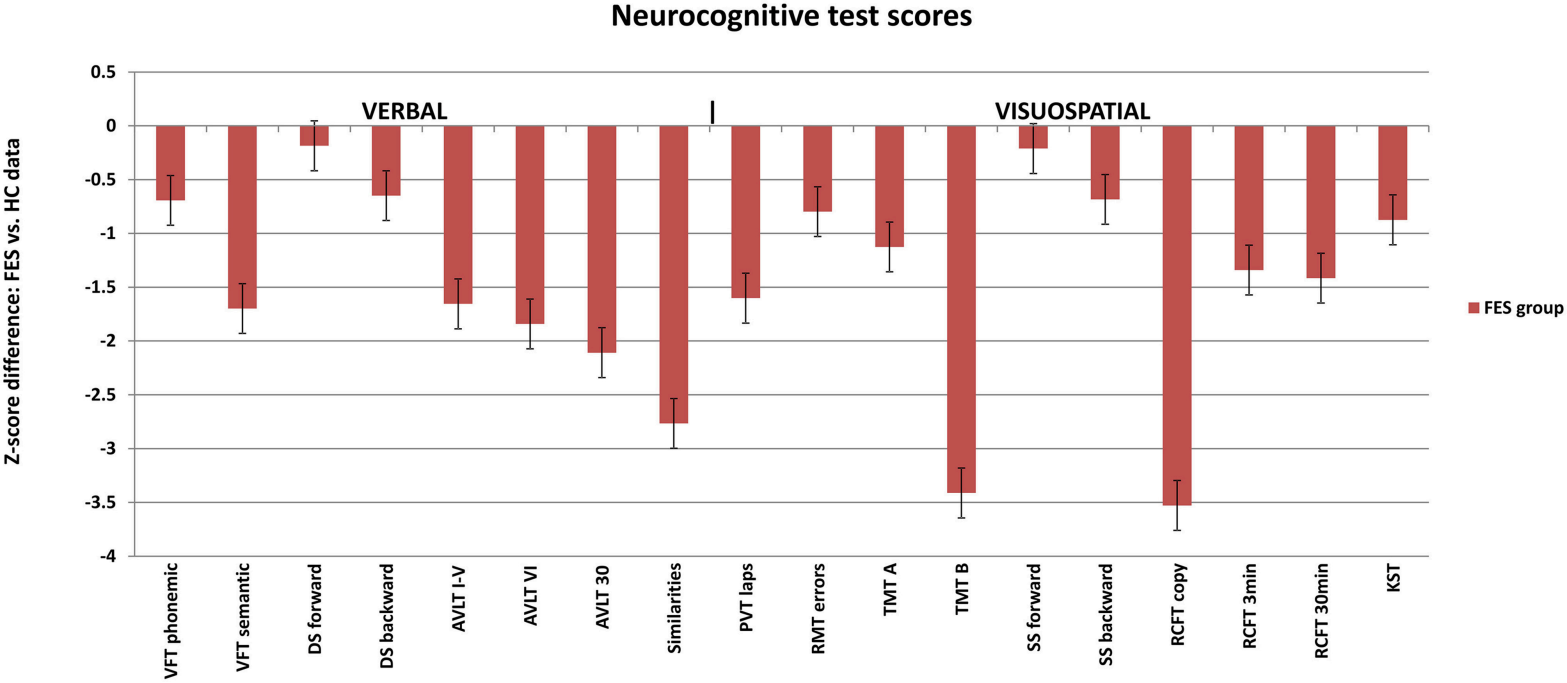
**Neuropsychological profile of FES subjects calculated in z-scores for individual test parameters relative to the healthy controls (average Z-score = 0)**. Selected test results are divided into verbal (left) and visuospatial (right) tests. FES, first psychotic episodes of schizophrenia spectrum disorder; HC, healthy controls; VFT, Verbal Fluency Test; AVLT, Auditory Verbal Learning Test; DS, Digit span; PVT, Perceptual Vigilance Test; TMT, Trail Making Test; RMT, Money Road-Map Test; RCFT, Rey-Osterrieth Complex Figure; SS, Spatial span; KST, Key Search Test.

As a result of matching the participants on an individual basis, no significant group differences in age and education were observed (Figure 1 and Table 1). In each group, there were 16 participants with a higher education level (university studies) and 20 with lower level of education.

Group differences in the results of neuropsychological tests were significant in most of the applied VIS and VERB measures, when analyzed from raw scores using the non-parametric Mann–Whitney *U*-test (for more details see Tables 3A,B).

**Table 3:** **Group differences in (A) Visuospatial and (B) Verbal neurocognitive tests**.

**Neurocognitive assessment**	**FES raw scores (*N* = 36)**				**HC raw scores (*N* = 36)**				**Mann–Whitney *U***	***p*-value**
	**mean**	**SE**	**median**	**SD**	**mean**	**SE**	**median**	**SD**		
**(A) VISUOSPATIAL PERFORMANCE**										
TMT–*A*	38.3	2.1	35.5	12.5	27.8	1.6	26.5	9.4	306.5	<0.001^***^
TMT–*B*	93.5	7.6	84.5	45.4	50.6	2.1	49.5	12.8	181	<0.001^***^
Ratio B/A	2.5	0.1	2.3	0.8	1.9	0.1	1.9	0.5	417	<0.01^**^
RCFT–copy	32.5	0.5	33.5	3.0	35.6	0.1	36	0.9	201	<0.001^***^
RCFT–3 min	18.8	1.1	22.0	6.5	25.7	0.9	26	5.2	234	<0.001^***^
RCFT–30 min	19.3	1.1	19.8	6.4	25.8	0.8	26	4.6	237	<0.001^***^
Recognition (errors)	4.6	0.4	4.5	2.1	3.6	3.0	0.3	1.9	360	0.073
KST	11.9	0.6	12.5	3.4	13.2	0.2	13	1.5	532.5	0.188
RMT–number of errors/32	3.1	0.6	2	3.6	1.2	0.4	0	2.4	394	0.003^**^
RMT A	0.3	0.1	0	0.8	0.1	0.1	0	0.5	628	0.661
RMT B	1.6	0.3	1	1.8	0.8	0.3	0	1.7	457.5	0.021^*^
RMT C	1.2	0.3	1	1.7	0.3	0.1	0	0.8	385	<0.001^***^
SS total	16.3	0.5	17	3.1	17.6	0.4	18	2.3	460	0.147
SS forward	8.6	0.3	9	1.6	8.9	0.2	9	1.4	535	0.592
SS backward	7.8	0.4	8	2.1	8.7	0.2	9	1.5	429	0.063
PVT–average response speed	337.3	8.7	321	44.5	305.5	8.0	294.5	40.8	189.5	0.007^**^
PVT–correct	66.3	1.8	70	10.0	70.7	0.8	72	4.9	415	0.068
PVT–comissions	1.3	0.4	0	2.5	0.9	0.2	0	1.3	541	0.791
PVT–lapses	7.9	1.6	4	9.1	2.1	0.6	1	3.6	279.5	<0.001^***^
**(B) VERBAL PERFORMANCE**										
AVLT—I-V	47.8	1.8	48.0	10.8	59.9	1.2	61.5	7.5	231.5	<0.001^***^
AVLT—3 min	9.3	0.4	9.0	2.5	13.1	0.3	14.0	2.1	157.5	<0.001^***^
AVLT—30 min	8.6	0.5	8.0	3.0	13.1	0.4	14.0	2.2	137.5	<0.001^***^
AVLT–repetitions	6.0	0.8	4.5	5.0	1.9	0.4	1.0	2.2	302.5	<0.001^***^
AVLT–confabulations	2.0	0.4	1.0	2.5	1.1	0.3	1.0	1.6	543.5	0.214
VFT phonemic	41.8	2.2	41	12.9	48.9	1.7	48.5	10.4	395.5	0.004^**^
VFT semantic	19.7	1.0	18.5	5.6	28.9	0.9	28.0	5.5	120	<0.001^***^
DS total	14.8	0.7	14.5	4.5	17.0	0.7	17.0	3.9	450	0.025^*^
DS forward	9.3	0.4	8.5	2.2	9.7	0.4	9	2.3	573	0.392
DS backward	5.2	0.4	6	2.1	7.4	0.4	7.5	2.3	397	0.004^**^
Similarities	22.9	0.8	23	4.9	28.8	0.4	29	2.2	165	<0.001^***^

SE, standard error of the mean; SD, standard deviation. For abbreviations of individual methods see Table 1. TMT, Trail Making Test; RCFT, Rey-Osterrieth Complex Figure; KST, Key Search Test; RMT, Money Road-Map Test (A, B, C—type of errors); SS, Spatial Span; PVT, Perceptual Vigilance Test; AVLT, Auditory Verbal Learning Test; VFT, Verbal Fluency Test; DS, Digit Span. Significance level:

* *p < 0.05*,

** *p < 0.01*,

*** *p < 0.001*.

**Figure 2:**
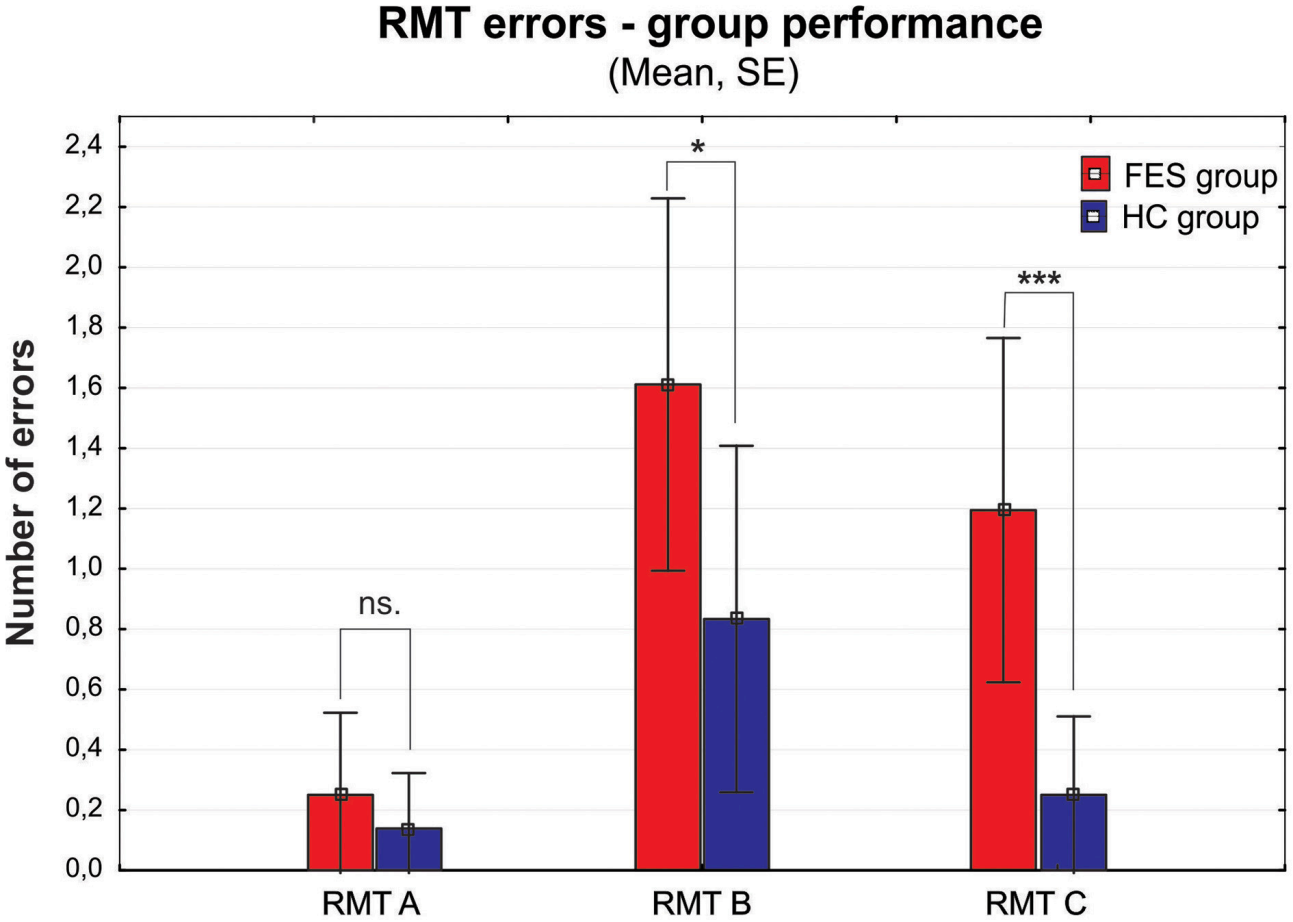
**Group performance presented as number of errors on the Money Road Map Test (RMT) (mean ± standard error)**. Errors are divided into three different types according to the rotation toward subjective perspective of the tested subject (A–less than 70° deviation from subject's heading; B–90°; C–more than 110°). FES group, first psychotic episodes of schizophrenia spectrum disorders; HC group, healthy controls. Significance; ns, non-significant; *p < 0.05, ***p < 0.001.

The group of FES subjects showed significantly lower VIS performance on all three parts of the Rey-Osterrieth Complex Figure Test (RCFT-copy, RCFT-3, RCFT-30). The FES group was also slower in the Trail Making Test part A and part B. The average speed of FES subject responses in the PVT was lower and they also had a higher number of lapses (with reaction times above 500 ms). The FES subjects also made more errors on the Road Map Test (RMT). In addition, splitting of individual turns in the RMT (according to Marková et al., 2015) to three possible error types showed that schizophrenia subjects tend to fail more in the turns demanding mental rotation of the spatial scene (turns B and C) compared to the turns that demand no or very small mental rotation (see Figure 2). We found no significant differences in the raw scores of the KST from the BADS battery. In contrast to the Digit Span (DS) test, the SS test did not show any significant group differences. However, the difference observed in the backward score was approaching the significance level (*p* = 0.063).

VERB performance was significantly impaired in most of the VERB measures as follows: verbal learning and delayed recall in the Verbal Learning Test (AVLT); the phonemic and semantic Verbal Fluency (VFT), abstract executive functions in the Similarities (Sim) and working memory in the DS task. No difference was observed in the immediate recall and attention performance in DS forward.

### Regression model of PANSS effect on performance in visuospatial and verbal tests in FES

First set of stepwise multiple regression analysis models of psychiatric symptomatology measured using PANSS (predictors: PANSS-P, PANSS-N, PANSS-G) showed no effect of symptomatology on the performance in individual visuospatial and verbal tests (dependent variables), except for the performance in one single test—the Trail Making Test part B. Two of the three predictors from the regression model performed by stepwise method were significant: PANSS-N and PANSS-G. They explained more than 40% of performance variability in the TMT-B test (see Table 4). No effect of individual PANSS scores was identified on cumulative VERB and VIS scores.

**Table 4:** **Regression model of PANSS effect (predictor) on performance in TMT B (dependent variable)**.

**Dependent variable *TMT–B***	**Adjusted *R*^2^**	**SE of the estimate**	**Durbin-Watson**	***B***	***SE***	***Beta***	***F***	***p***
**Predictor variable (Model):**	**0.409**	**35.1850**	**1.850**				**12.4**	<**0.001**
*(Constant)*				112,244	29,361			0.001
*PANSS-N*				3.766^***^	0.850	0.599		<0.001
*PANSS-G*				−2.968^**^	1.034	−0.388		0.007
Excluded:								
PANSS-P						0.006		0.968

Stepwise multimodal linear regression in FES subjects (N = 34); PANSS, Positive and Negative Symptoms Scale (N, negative; G, general; P, positive); TMT-B, Trail Making Test –part 2; Adjusted R^2^, explained variability; B, Unstandardized Coefficients beta; SE, Standard error; Beta, Standardized beta coefficient; Significance:

** *p < 0.01*,

*** *p < 0.001*.

### Regression model of CPZ level effect on performance in visuospatial and verbal tests in FES

We did not observe any significant influence of medication (CPZ) on the performance in any of the visuospatial tests using the linear regression analysis. We identified negative effect of medication dosage (*B* = −0.040, *SE* = 0.017, Beta = −0.373) only in one verbal test—the phonemic Verbal Fluency Test (*N* = 35). CPZ explained 11% of performance variability (*SE* = 12.253, Durbin-Watson = 2.247; *F* = 5.338, *p* < 0.05). This observation would not survive Bonferroni adjustments (*p* = 0.027). No effect of CPZ level was identified on cumulative VERB and VIS scores.

### Regression model of clinical and neuropsychological factors on global functioning in FES

In order to analyze the possible effect of verbal and nonverbal cognitive performance on global functioning (measured on the GAF scale), we performed a multiple regression analysis with the following predictors: PANSS-P, PANSS-N, PANSS-G, CPZ level, and cumulative VIS and VERB scores (see Table 5). Only two of the independent variables, the severity of positive symptoms (PANSS-P score) and verbal functioning (cumulative VERB score), showed a significant effect on global functioning. Together, these variables explained more than 60% of GAF variability.

**Table 5:** **Regression model of clinical and neuropsychological variables (predictors) on global functioning measured by GAF (dependent variable)**.

**Dependent variable: *GAF***	**Adjusted *R*^2^**	**SE of the estimate**	**Durbin-Watson**	***B***	***SE***	***Beta***	***F***	***p***
**Predictor variable (Model):**	**0.587**	**11.407**	**1.954**				**18.775**	<**0.001**
(Constant)				104.853	6.971			<0.001
*PANSS–P*				−2.153^***^	0.389	−0.712		<0.001
*VERB*				0.898^*^	0.388	0.298		0.030
*Excluded:*								
*PANSS–N*						−0.168		0.248
*PANSS–G*						−0.105		0.461
*CPZ*						0.044		0.754
*VIS*						0.093		0.603

Stepwise multimodal linear regression in FES subjects (N = 26); GAF, Global Assessment of Functioning; PANSS, Positive and Negative Syndrome Scale (P, positive; N, negative; G, general); CPZ, chlorpromazine equivalents; VERB, cumulative score for verbal tests; VIS, cumulative score for visuospatial tests; Adjusted R^2^, explained variability; B, Unstandardized Coefficients beta; SE, Standard error; Beta, Standardized beta coefficient; Significance:

* *p < 0.05*,

*** *p < 0.001*.

### Regression model of clinical and neuropsychological factors on quality of life (QOL) in FES

The same model of clinical and neuropsychological parameters was applied in the stepwise multiple regression analysis that identified significant effect of severity of negative symptoms (PANSS-N score), verbal functioning (cumulative VERB score) and positive symptoms (PANSS-P score) on perceived quality of Physical health (domain 1) in FES. These predictors together explained nearly 50 % of the observed variability (for details see Table 6). The QOL domain Psychological health (domain 2) was found to be affected by the overall VERB functioning (see Table 7).

**Table 6:** **Regression model of clinical and neuropsychological variables (predictors) on perceived quality of Physical health (dependent variable)**.

**Dependent variable: *Physical health***	**Adjusted *R*^2^**	**SE of the estimate**	**Durbin-Watson**	***B***	***SE***	***Beta***	***F***	***p***
**Predictor variable (Model):**	**0.485**	**1.8198**	**1.753**				**8.234**	**0.001**
(Constant)				17.064	1.202			<0.001
*PANSS–N*				−0.162^**^	0.050	−0.520		0.004
*VERB*				0.173^*^	0.067	0.405		0.017
*PANSS–P*				0.150*	0.066	0.353		0.033
*Excluded:*								
*PANSS–G*						−0.079		0.628
*CPZ*						−0.172		0.354
*VIS*						−0.206		0.385

Stepwise multimodal linear regression (N = 24); Physical health, domain 1 in WHOQOL-BREF; PANSS, Positive and Negative Syndrome Scale (P, positive; N, negative; G, general); CPZ, chlorpromazine equivalents; VERB, cumulative score for verbal tests; VIS, cumulative score for visuospatial tests; Adjusted R^2^, explained variability; B, Unstandardized Coefficients beta; SE, Standard error; Beta, Standardized beta coefficient; Significance:

* *p < 0.05*,

** *p < 0.01*.

**Table 7:** **Regression model of clinical and neuropsychological variables (predictors) on perceived quality of Psychological health (dependent variable)**.

**Dependent variable: *Psychological health***	**Adjusted *R*^2^**	**SE of the estimate**	**Durbin-Watson**	***B***	***SE***	***Beta***	***F***	***p***
**Predictor variable (Model):**	**0.144**	**2.235**	**1.803**				**4.859**	**0.038**
(Constant)				16.214	1.013			<0.001
*VERB*				0.173^*^	0.079	0.425		0.038
*Excluded:*								
*PANSS–P*						0.031		0.879
*PANSS–N*						−0.235		0.250
*PANSS–G*						0.016		0.934
*CPZ*						−0.087		0.691
*VIS*						−0.107		0.728

Stepwise multimodal linear regression (N = 27); Psychological health, domain 2 in WHOQOL-BREF; PANSS, Positive and Negative Syndrome Scale (P, positive; N, negative; G, general); CPZ, chlorpromazine equivalents; VERB, cumulative score for verbal tests; VIS, cumulative score for visuospatial tests; Adjusted R^2^, explained variability; B, Unstandardized Coefficients beta; SE, Standard error; Beta, Standardized beta coefficient; Significance:

* *p < 0.05*.

No significant effect of clinical and neuropsychological parameters was observed in the evaluated quality of Social relationships (domain 3). In contrast, the Environment quality (domain 4) appeared to be affected mostly by one significant parameter, the cumulative VIS score performance. This factor explained together with CPZ level around 27% of the observed variability in domain 4 (see Table 8). However, worse Environment QOL was predicted by better VIS functioning and higher AP dosage.

**Table 8:** **Regression model of clinical and neuropsychological variables (predictors) on perceived quality of Environment (dependent variable)**.

**Dependent variable: *Environment***	**Adjusted *R*^2^**	**SE of the estimate**	**Durbin-Watson**	***B***	***SE***	***Beta***	***F***	***p***
**Predictor variable (Model):**	**0.278**	**1.9072**	**1.986**				**5.425**	**0.013**
(Constant)				15.946	1.404			<0.001
*VIS*				−0.107**	0.037	−0.533		0.008
*CPZ*				−0.008^*^	0.004	−0.392		0.043
Excluded:								
*PANSS—P*						−0.068		0.711
*PANSS—N*						−0.141		0.495
*PANSS—G*						0.224		0.251
*VERB*						−0.109		0.720

Stepwise multimodal linear regression (N = 24); Environment, domain 4 in WHOQOL-BREF; PANSS, Positive and Negative Syndrome Scale (P, positive, N, negative, G, general); CPZ, chlorpromazine equivalents; VERB, cumulative score for verbal tests; VIS, cumulative score for visuospatial tests; Adjusted R^2^, explained variability; B, Unstandardized Coefficients beta; SE, Standard error; Beta, Standardized beta coefficient; Significance:

* *p < 0.05*.

## Discussion

### Differences in cognitive performance between FES and control group

Our study confirmed the presence of a deficit in visuospatial cognitive abilities in a sample of first psychotic episode of schizophrenia spectrum disorder subjects, when compared with matched group of healthy controls. Similar pattern of deficit was also observed in verbal functions. This result is in accordance with previous studies, both in the first episodes and chronic SZ subjects (e.g., Green et al., 2004; Fioravanti et al., 2005). First, we selected the PVT as an indicator of vigilance and response speed, as deficits in these abilities could affect performance in other applied measures. Our sample of FES subjects demonstrated deficit in vigilance and response speed on the PVT task. However, this deficit ranged between none and moderate (SD 0–1.5), with more severe deficits observed in other cognitive measures. Nevertheless, we suggest future visuospatial studies apply such attentional measure as a covariate factor in order to clarify how vigilance and response speed may affect tested visuospatial abilities.

Deficit on visuospatial functions was manifested in perceptual organization abilities (copy in the Rey-Osterrieth Complex Figure Test- RCFT) and in the delayed recall of visuospatial information (RCFT after 3 and 30 min), while the recognition (RCFT recognition) of visuospatial material was comparable with the performance of HC. This finding was characterized by a reduced number of recalled details but not by an increased rate of forgetting. This is consistent with a disturbance in encoding or retrieval from memory but no deficit in information storage (Rodriguez, 2012). The problems in recall might be further compounded by a primary deficit in visual scanning abilities or limited analysis of the visual fields, leading to the omission of significant details and even whole sections of the figure (Golden et al., 2002).

Trail Making Test (TMT-A and B) also showed significantly slower processing speed, impaired visuomotor tracking and switching ability. Although the performance on the TMT might be perceived as partly language dependent, since the sequence of numbers or numbers and letters has a verbal component, participants' visuospatial tracking ability is the main cognitive domain assessed by this task. The higher ratio of TMT B/A also pointed out a deficit in executive control function (Lezak et al., 2012). The repeated finding of significantly impaired TMT A and B performance in SZ (Heinrichs and Zakzanis, 1998), complemented by the TMT ratio B/A deficit, suggests an independent deficit in both processing speed and switching ability. Similar to the RCFT, the problems on both these functions might be further compounded by a primary deficit in visual scanning abilities. Future research in this area is needed.

Interesting results were found in the Money Road Map Test (RMT), which to our knowledge has not been previously used in FES patients. The higher number of total errors shows deficit in left/right direction sense, and additionally in perspective taking abilities (Schultz, 1991; Marková et al., 2015). These abilities are suggested as the main solution strategy in RMT. The lower RMT performance in FES group is therefore even more apparent, if the individual intersections are divided into three types according to their perspective taking demands, which is essential in order to respond in the left/right condition. While the number of errors in condition A (no change in perspective) is not significantly disrupted in FES, both conditions B and C (90 and more degrees deviations requiring perspective taking) showed significant impairment. On top of that, the number of errors increased with the growing degree of deviation (from condition A–C), similarly to the mental rotation abilities described previously (e.g., de Vignemont et al., 2006). This finding deserves further investigation and standardization of the method that could lead to a wider usage of this test in SZ clinical research. Moreover, due to its spatial characteristic the test could also be very useful in comparative studies of SZ.

The visuospatial executive planning ability, measured with the Key Search Test (KST), failed to show a deficit in FES subjects. Impairment in KST was previously demonstrated in studies of chronic SZ (Evans et al., 1997; Ihara et al., 2003; Vargas et al., 2009). The fact that we evaluated first psychotic episode of schizophrenia spectrum patients in early remission could explain this contradictory finding. Ihara et al. (2003) showed connection between the KST performance and the severity of negative symptoms in chronic SZ subjects. The mild severity of negative symptoms in our FES group could be responsible for the lack of significance found in KST. The lack of information about the symptomatology in the other two cited studies does not allow us to properly compare our findings. In addition, the fact that the differences were analyzed using the raw scores (0–16) could cause smaller sensitivity of the KST measure, as the recalculated profile scores (range 0–4) separate the performance more strictly into five categories. To test this presumption, we did an ex post facto analysis with raw scores transposed to profile scores, which indeed led to significant disadvantage of the FES group (*U* = 445; *p* = 0.023).

Verbal abilities showed a deficit in conceptualization and executive functions (semantic Verbal Fluency Test and Similarities), and in verbal learning and delayed recall (AVLT). It would be interesting to also analyze the verbal recognition pattern and compare it to the results of visuospatial recognition. Studies addressing this topic in SZ showed that the verbal recognition is preserved (see Fiszdon et al., 2008). However, because of some missing data in verbal recognition of HC group we were unable to complete this comparison. We are aware that this is a limitation of the present study. The significant increase in the number of repetitions suggests that SZ subjects have difficulties in self-monitoring and tracking abilities that are the key in the retrieval process (Lezak et al., 2012).

The verbal and visuospatial measures (DS and SS) of immediate recall and working memory (WM) showed similar patterns in FES (see Figure 1). No deficit was observed on either the verbal or visuospatial tasks assessing immediate recall and attention (DS and SS forward). Even though the deficit in verbal WM (DS backward) was stronger than in the spatial WM, there was a trend of significance in SS backward (*p* = 0.06). Moreover, the pattern of deviation in both tests was similar (see Figure 1). One of the reasons for the lack of significance in SS backward could be the smaller size of the study sample producing reduced power size. Other reason might be the use of a computerized version of the task. Because the computerized version has not been validated, its sensitivity can be questioned. However, computerized version of SS and its alternative, the Corsi block tapping test, are commonly used in clinical studies (e.g., Kessels et al., 2000; or studies reported in Lezak et al., 2012), and they bring the advantage of administration reliability independent of examiner bias. Despite the lack of significance in the SS backward, the finding of impaired TMT-B (switching) supports the assumption of visuospatial WM deficit in SZ.

### Effect of symptomatology on cognitive functioning in FES

In agreement with more recent reviews and empirical reports (Andreasen et al., 2005; Keefe and Fenton, 2007; Ventura et al., 2009), we confirm the absence of relations between the symptoms severity and standard cognitive measures, except for performance on the Trail Making Test (TMT-B). TMT-B performance was negatively affected by negative symptoms and positively affected by general symptomatology. No effect of positive symptoms was identified. Current literature describes a strong to moderate association of cognitive functioning and negative symptoms (O'Leary et al., 2000), whereas positive symptoms and cognitive performance are usually independent in SZ (Addington et al., 1991; Rossi et al., 1997; Andreasen et al., 2005). The finding that better TMT-B performance was predicted by worse general symptomatology could be explained by the fact that the PANSS scale had higher inter-rater variability, particularly in the negative and general symptomatology, which could generate distortion in our findings. Indeed, our FES group showed higher scores especially in some general symptoms (not reported in detail), such as the item G4 (tension), which was previously identified as more difficult for some raters (Khan et al., 2013). Despite the single observation in TMT-B, in general, the cognitive performance showed to be independent of clinical symptoms.

### Effect of pharmacological treatment on cognitive functioning in FES

In agreement with other studies (Jones et al., 2006; Keefe et al., 2007; Lewis and Lieberman, 2008), we found no effect of atypical antipsychotic medication (antipsychotic dosage calculated in CPZ equivalents) on visuospatial or verbal performance, except the phonemic verbal fluency performance. There was a negative effect of CPZ on phonemic Verbal Fluency Test; however, this result became non-significant after Bonferroni correction. Our study implies that the impairment in visuospatial functions is independent of the dosage of neuroleptic medication.

### Clinical factors and neurocognition, and their effect on global functioning in schizophrenia spectrum disorder

In our FES group we did not find any associations between GAF and VIS functions. The fact that the only visuospatial measure that correlated with the GAF score was the Trail Making Test B^1^ could be responsible for this negative finding. Moreover, the strong association between the negative and general symptoms toward TMT-B performance described earlier (see Section Effect of Symptomatology on Cognitive Functioning in FES) suggests that VIS performance, moderated by the symptomatology, might not survive the regression analysis as an independent predictor. Another possible explanation for this finding is the fact that GAF scale was constructed as a measure of psychosocial disability in relation to symptomatology, rather than neurocognition (Jones et al., 1995; Roy-Byrne et al., 1996). Thus, more specific neurocognitive functions, such as VIS, might not be captured. On the other hand, GAF was positively affected by VERB functions (cumulative VERB score). However, VERB functioning was only an accompanying factor of the main negative effect produced by positive symptoms. The effect of negative symptoms reported in previous studies (Gaite et al., 2005) was not identified as significant in our FES sample. We assume that positive symptoms might have a more pronounced negative effect on the functioning of individuals' in our FES sample than negative and general symptoms, due to the early remission state.

In order to compare our results with previous studies, we used the GAF as a scale of functioning recommended as a mandatory control assessment by EGOFORS (European Group On Functional Outcomes and Remission in Schizophrenia) initiative (Peuskens and Gorwood, 2012). However, in our opinion, a more ecologically valid scale to measure functioning in relationship to individual neurocognitive domains is needed.

### Clinical factors and neurocognition, and their effect on quality of life in schizophrenia spectrum disorder

According to our results, the quality of life seems to be related more to verbal than visuospatial cognitive measures. Two of the four domains of WHOQOL-BREF (Physical and Psychological health) were positively associated with overall VERB performance, whereas only one domain (Environment) was related to overall VIS functioning and this association was negative. Better cumulative VIS score was associated with worse environment quality (health services, leisure time activities, etc.). Some previous studies have reported similar counterintuitive negative correlations (Prouteau et al., 2005; Fiszdon et al., 2008; Narvaez et al., 2008). This negative relation between QOL and neurocognition is often explained with a lack of insight (Prouteau et al., 2005; Narvaez et al., 2008) or with an overestimation of the level of disability due to present depressive symptoms (Bowie et al., 2007). We do not attempt to interpret this negative relation in terms of insight, as some other possible moderators might attenuate the relationship between neurocognition and QOL. However, we are aware of this discrepancy and we suggest that future research is needed in order to clarify the character of such puzzling results.

The WHOQOL-BREF domain of Social relationships was not associated with any of the clinical or neurocognitive measures. We believe that this domain might not fully reflect quality of social relationships. This domain includes only three questions that report on the quality of social relations, sexual life, and social support. These items do not cover all aspects of interpersonal relationships. Moreover, the item “Friend's support” was reported to be less relevant for the younger population assessed also in our study (Dragomirecká and Bartoňová, 2006a). WHOQOL-BREF might therefore not be a suitable tool for the measurement of social QOL in such a specific population.

Our choice of QOL measure, the WHOQOL-BREF, likely also played a role in the obtained results. In general, research findings on the relationship of neurocognition and QOL are very heterogeneous and often report weak associations between these two constructs (Heslegrave et al., 1997; Aksaray et al., 2002; Fiszdon et al., 2008). One issue that has to be considered is how other QOL questionnaires address cognition in individual items. For example, only one question of the WHOQOL-BREF specifically concentrates on the cognitive functioning. When we ex post facto analyzed the correlation between this item (Q7, quality of concentration in Psychological health domain) and individual cognitive measures, we found a strong relationship toward several cognitive tests, both VIS and VERB (mostly related to processing speed, memory and executive functions)^2^. This is in agreement with previous studies that highlighted the role of executive functions (e.g., Fiszdon et al., 2008; Matsui et al., 2008) and memory domains as the most representative measures related to QOL. If QOL questionnaires were more focused on cognitive functioning, we believe that the contradictory findings could be reduced.

In terms of clinical symptomatology, out of the four WHOQOL-BREF domains only Physical health appeared to be significantly affected by psychiatric symptoms. As expected, cognitive performance was not the only factor affecting subjective quality of physical health; the severity of the negative and positive symptoms obviously had some impact as well. Meta-analysis by Eack and Newhill (2007) described the strongest, but still small, association of Physical health QOL to general symptoms. However, our study found no such association. An explanation for the differences between our results and the previous findings can be the fact that we assessed first psychotic episode in schizophrenia spectrum patients and that the length of illness might moderate the relationship between symptoms and QOL (Eack and Newhill, 2007).

### Concluding remarks

The studies addressing effect of cognitive deficit on global functioning or QOL are common in current research of SZ. However, studies that address these findings in first psychotic episode of schizophrenia spectrum disorder and which include complex verbal and visuospatial cognitive assessment are quite rare. We addressed the need for such research in the present study.

The results of the present study confirmed a deficit of visuospatial functions in FES. This deficit is independent of antipsychotic medication and clinical symptoms. Both global functioning and quality of life were shown to be more related to verbal than to visuospatial functions. Given the findings of negative or missing effect of visuospatial deficit on WHOQOL-BREF and GAF, the accuracy of these measures to evaluate the impact of global cognitive deficit on everyday life in schizophrenia could be questioned. We suggest the need for further investigation of the association of QOL questionnaires and GAF scale to cognitive functioning. Finally, according to our findings, the deficit in executive and memory domains is the most pronounced in the FES group. We suggest that these two domains may contribute to the cumulative cognitive performance affecting QOL and GAF scores. Further research needs to clarify this assumption.

### Limitations of the study and future directions

There are several limitations of the present study that warrant discussion. First, the results of the study could be tempered by the small size of the sample and consequent reduction in power size. Despite the smaller number of participants, we were able to demonstrate the deficit in both visuospatial and verbal cognitive functioning in schizophrenia, and the relationship of these abilities toward global functioning and quality of life. In order to reveal possible effects of other variables on these relationships (such as demography and subtypes of schizophrenia) and to identify individual cognitive domains affecting QOL and GAF, a larger sample size is needed.

Second, the issue of cross-sectional vs. longitudinal studies in this area is important. The present study, although cross-sectional, has identified some specific effects that can be examined over a longer time period. The next step in this research is, therefore, to track the longitudinal effect of visuospatial functions on global functioning and QOL in schizophrenia. We are currently conducting a follow-up assessment in our study group 1 year after their first hospitalization to measure the cognitive functioning in full remission state. Moreover, studies comparing FES with chronic schizophrenia patients are limited. To address this limitation we are assessing a chronic SZ sample in order to analyze the influence of the illness duration on relations between neurocognition, QOL, and GAF.

Third, the fact that the verbal and visuospatial neuropsychological tests were not always matched in terms of the measured cognitive domain, and for psychometric parameters, might be another possible limiting factor. In addition, not all test methods are validated in schizophrenia and some of them are not standardized for Czech population either. Measures validated in schizophrenia population might be expected to be more sensitive when capturing a degree of deficit. We are also aware of the fact that we don't cover all the functions of each cognitive domain. For example we did not include verbal recognition in our analysis; therefore the encoding ability could not be assessed as clearly as in the visuospatial domain. More specific and detailed visuospatial assessment is necessary in order to cover all domains that can be related to functional outcome and QOL in schizophrenia spectrum disorders.

Fourth, measures of QOL and global functioning applied in this study could be limited in terms of their association with cognitive functioning in SZ. It would be very helpful to compare them to other methods that might be more related to neurocognition. For example, the Social and Occupational Functional Assessment Scale (SOFAS) could provide a better measurement of functional outcome that is not tied to symptomatology. In addition, only subjective QOL was measured in this study. Objective measures of QOL are needed in order to understand the complex relationships of psychosocial functioning and neurocognition in SZ. According to our results about relation of specific domains of QOL and GAF obtained in the ex post facto measures, we suggest that these associations should be further investigated in future research.

Finally, several neurotransmitter functions are affected by atypical antipsychotics and our study applied only the CPZ equivalent. Future study should also address other factors in order to analyze the effects of medication on cognitive abilities in greater detail.

## Author contributions

MR, KV, and IF designed the study and together with KD, FS, and ZK wrote the original protocol. IF, DL, LK, and KS recruited the participants, and they performed the neuropsychological assessment. IF and KV pre-processed the data and JP undertook the statistical analysis. MR supervised the study. MR and IF wrote the first draft of the manuscript. FS, KV, and KD contributed to data interpretation. All of the authors discussed the results and contributed to the final version of the paper and have approved it.

### Conflict of interest statement

The authors declare that the research was conducted in the absence of any commercial or financial relationships that could be construed as a potential conflict of interest.

## Footnotes

^1^Ex post facto analysis revealed that the GAF scale is significantly correlated only with two neurocognitive measures. Negative correlation was found in visual Trail Making Test B (*r* = −0.43, *p* < 0.001) and positive correlation in the semantic Verbal Fluency Test (*r* = 0.36, *p* < 0.01).

^2^Ex post facto analyzed relationship of Psychological health item Q7 (concentration) with individual test methods, showed positive correlation with the AVLT test in terms of learning (*r* = 0.572) and delayed memory (*r* = 0.508), Verbal Fluency (phonemic: *r* = 0.519; semantic: *r* = 0.457) and Spatial Span performance (total score: *r* = 0.479; backwards: *r* = 0.445), and similarly negative correlation with TMT processing time (part A: *r* = −0.432; B: *r* = −0.602).

## References

[B1] Addington J. Addington D. Maticka-Tyndale E. (1991). Cognitive functioning and positive and negative symptoms in schizophrenia. Schizophr. Res. 5, 123–134. 10.1016/0920-9964(91)90039-T 1931805

[B2] Aksaray G. Oflu S. Kaptanoglu C. Bal C. (2002). Neurocognitive deficits and quality of life in outpatients with schizophrenia. Prog. Neuropsychopharmacol. Biol. Psychiatry 26, 1217–1219. 10.1016/S0278-5846(02)00217-8 12452550

[B3] Andreasen N. C. Carpenter W. T. Jr. Kane J. M. Lasser R. A. Marder S. R. Weinberger D. R. (2005). Remission in schizophrenia: proposed criteria and rationale for consensus. Am. J. Psychiatry 162, 441–449. 10.1176/appi.ajp.162.3.441 15741458

[B4] Andreasen N. C. Pressler M. Nopoulos P. Miller D. Ho B. C. (2010). Antipsychotic dose equivalents and dose-years: a standardized method for comparing exposure to different drugs. Biol. Psychiatry 67, 255–262. 10.1016/j.biopsych.2009.08.040 19897178 PMC3677042

[B5] Arana G. W. (2000). An overview of side effects caused by typical antipsychotics. J. Clin. Psychiatry 61(Suppl. 8), 5–11. Available online at: http://www.psychiatrist.com/JCP/article/Pages/2000/v61s08/v61s08002a.aspx 10811237

[B6] Bilder R. M. Goldman R. S. Robinson D. Reiter G. Bell L. Bates J. A.. (2000). Neuropsychology of first-episode schizophrenia: initial characterization and clinical correlates. Am. J. Psychiatry 157, 549–559. 10.1176/appi.ajp.157.4.549 10739413

[B7] Bilder R. M. Turkel E. Lipschutz-Broch L. Lieberman J. A. (1992). Antipsychotic medication effects on neuropsychological functions. Psychopharmacol. Bull. 28, 353–366. 1363583

[B8] Bowie C. R. Twamley E. W. Anderson H. Halpern B. Patterson T. L. Harvey P. D. (2007). Self-assessment of functional status in schizophrenia. J. Psychiatr. Res. 41, 1012–1018. 10.1016/j.jpsychires.2006.08.003 17014866 PMC3634704

[B9] Bubeníková V. Votava M. Horáček J. Páleníček T. Dockery C. (2005). The effect of zotepine, risperidone, clozapine and olanzapine on MK-801-disrupted sensorimotor gating. Pharmacol. Biochem. Behav. 80, 591–596. 10.1016/j.pbb.2005.01.012 15820528

[B10] Butler P. D. Zemon V. Schechter I. Saperstein A. M. Hoptman M. J. Lim K. O.. (2005). Early-stage visual processing and cortical amplification deficits in schizophrenia. Arch. Gen. Psychiatry 62, 495–504. 10.1001/archpsyc.62.5.495 15867102 PMC1298183

[B11] Černochová D. Goldmann P. Král P. Soukupová T. Šnorek P. Havlůj V. (2010). Wechslerova Inteligenční škála Pro Dospělé WAIS-III. Praha: Hogrefe-Test Centrum.

[B12] Chaplin R. Barley M. Cooper S. J. Kusel Y. McKendrick J. Stephenson D.. (2006). The impact of intellectual functioning on symptoms and service use in schizophrenia. J. Intellect. Disabil. Res. 50, 288–294. 10.1111/j.1365-2788.2006.00837.x 16507033

[B13] Cocchi L. Bosisio F. Berchtold A. Orita A. Debbané M. Wood S. J.. (2009). Visuospatial encoding deficits and compensatory strategies in schizophrenia revealed by eye movement analysis during a working memory task. Acta Neuropsychiatr. 21, 75–83. 10.1111/j.1601-5215.2009.00369.x 25384566

[B14] Cummings J. L. Mega M. S. (2003). Visuospatial, visuoperceptual, and right hemisphere disturbances, in Neuropsychiatry and Behavioral Neuroscience, eds Cummings J. L. Mega M. S. (NewYork, NY: Oxford University Press), 114–127.

[B15] de Vignemont F. Zalla T. Posada A. Louvegnez A. Koenig O. Georgieff N.. (2006). Mental rotation in schizophrenia. Conscious. Cogn. 15, 295–309. 10.1016/j.concog.2005.08.001 16182569

[B16] Dinges D. F. Orne M. T. Orne E. C. (1985). Assessing performance upon abrupt awakening from naps during quasi-continuous operations. Behav. Res. Methods Instrum. Comput. 17, 37–45. 10.3758/BF03200895

[B17] Doniger G. M. Silipo G. Rabinowicz E. F. Snodgrass J. G. Javitt D. C. (2001). Impaired sensory processing as a basis for object-recognition deficits in schizophrenia. Am. J. Psychiatry 158, 1818–1826. 10.1176/appi.ajp.158.11.1818 11691687

[B18] Dragomirecká E. Bartoňová J. (2006a). Dotazník kvality života Světové zdravotnické organizace WHOQOL-BREF: Psychometrické vlastnosti a první zkušenosti s českou verzí (The World Health Organization quality of life assessment WHOQOL_BREF. Psychometric properties and first experience with czech version). Psychiatrie 10, 144–149. Available online at: http://www.tigis.cz/images/stories/psychiatrie/2006/03/02_dragomirec_psych_3-06.pdf

[B19] Dragomirecká E. Bartoňová J. (2006b). WHOQOL-BREF, WHOQOL-100: World Health Organization Quality of Life Assessment: příručka pro uživatele české verze dotazníků kvality života Světové zdravotnické organizace. Praha: Psychiatrické centrum Praha.

[B20] Eack S. M. Newhill C. E. (2007). Psychiatric symptoms and quality of life in schizophrenia: a meta-analysis. Schizophr. Bull. 33, 1225–1237. 10.1093/schbul/sbl071 17204532 PMC2632363

[B21] Evans J. J. Chua S. E. McKenna P. J. Wilson B. A. (1997). Assessment of the dysexecutive syndrome in schizophrenia. Psychol. Med. 27, 635–646. 10.1017/S0033291797004790 9153684

[B22] Fajnerová I. Rodriguez M. Levčík D. Konrádová L. Mikoláš P. Brom C.. (2014). A virtual reality task based on animal research - spatial learning and memory in patients after the first episode of schizophrenia. Front. Behav. Neurosci. 8:157. 10.3389/fnbeh.2014.00157 24904329 PMC4034703

[B23] Fiszdon J. M. Choi J. Goulet J. Bell M. D. (2008). Temporal relationship between change in cognition and change in functioning in schizophrenia. Schizophr. Res. 105, 105–113. 10.1016/j.schres.2008.06.010 18657398

[B24] Fioravanti M. Carlone O. Vitale B. Cinti M. E. Clare L. (2005). A meta-analysis of cognitive deficits in adults with a diagnosis of schizophrenia. Neuropsychol. Rev. 15, 73–95. 10.1007/s11065-005-6254-9 16211467

[B25] Folley B. S. Astur R. Jagannathan K. Calhoun V. D. Pearlson G. D. (2010). Anomalous neural circuit function in schizophrenia during a virtual Morris water task. Neuroimage 49, 3373–3384. 10.1016/j.neuroimage.2009.11.034 19948225 PMC2818580

[B26] Gaite L. Vázquez-Barquero J. L. Herrán A. Thornicroft G. Becker T. Sierra-Biddle D.. (2005). Main determinants of global assessment of functioning score in schizophrenia: a European multicenter study. Compr. Psychiatry 46, 440–446. 10.1016/j.comppsych.2005.03.006 16275211

[B27] Golden C. J. Espe-Pfeifer P. Wachsler-Felder J. (2002). Neuropsychological Interpretation of Objective Psychological Tests. Fort Lauderdale, FL: Kluwer Academic Publishers; Nova Southeastern University.

[B28] Green M. F. Nuechterlein K. H. Gold J. M. Barch D. M. Cohen J. Essock S.. (2004). Approaching a consensus cognitive battery for clinical trials in schizophrenia: the NIMH-MATRICS conference to select cognitive domains and test criteria. Biol. Psychiatry 56, 301–307. 10.1016/j.biopsych.2004.06.023 15336511

[B29] Hanlon F. M. Weisend M. P. Hamilton D. A. Jones A. P. Thoma R. J. Huang M.. (2006). Impairment on the hippocampal-dependent virtual Morris water task in schizophrenia. Schizophr. Res. 87, 67–80. 10.1016/j.schres.2006.05.021 16844347

[B30] Harvey P. D. Sabbag S. Prestia D. Durand D. Twamley E. W. Patterson T. L. (2012). Functional milestones and clinician ratings of everyday functioning in people with schizophrenia: overlap between milestones and specificity of ratings. J. Psychiat. Res. 46, 1546–1552. 10.1016/j.jpsychires.2012.08.018 22979993 PMC3485423

[B31] Heinrichs R. W. Zakzanis K. K. (1998). Neurocognitive deficit in schizophrenia: a quantitative review of the evidence. Neuropsychology 12, 426–445. 10.1037/0894-4105.12.3.426 9673998

[B32] Heslegrave R. J. Awad A. G. Voruganti L. N. (1997). The influence of neurocognitive deficits and symptoms on quality of life in schizophrenia. J. Psychiatry Neurosci. 22, 235–243. 9262045 PMC1188864

[B33] Houthoofd S. A. Morrens M. Sabbe B. G. (2008). Cognitive and psychomotor effects of risperidone in schizophrenia and schizoaffective disorder. Clin. Ther. 30, 1565–1589. 10.1016/j.clinthera.2008.09.014 18840365

[B34] Ihara H. Berrios G. E. McKenna P. J. (2003). The association between negative and dysexecutive syndromes in schizophrenia: a cross-cultural study. Behav. Neurol. 14, 63–74. 10.1155/2003/304095 14757982 PMC5497557

[B35] Jones P. B. Barnes T. R. Davies L. Dunn G. Lloyd H. Hayhurst K. P.. (2006). Randomized controlled trial of the effect on quality of life of second- vs first-generation antipsychotic drugs in schizophrenia: Cost Utility of the Latest Antipsychotic Drugs in Schizophrenia Study (CUtLASS 1). Arch. Gen. Psychiatry 63, 1079–1087. 10.1001/archpsyc.63.10.1079 17015810

[B36] Jones S. H. Thornicroft G. Coffey M. Dunn G. (1995). A brief mental health outcome scale-reliability and validity of the Global Assessment of Functioning (GAF). Br. J. Psychiatry 166, 654–659. 10.1192/bjp.166.5.654 7620753

[B37] Kay S. R. Fiszbein A. Opler L. A. (1987). The positive and negative syndrome scale (PANSS) for schizophrenia. Schizophr. Bull. 13, 261–276. 10.1093/schbul/13.2.261 3616518

[B38] Keefe R. S. Bilder R. M. Davis S. M. Harvey P. D. Palmer B. W. Gold J. M.. (2007). Neurocognitive effects of antipsychotic medications in patients with chronic schizophrenia in the CATIE Trial. Arch. Gen. Psychiatry 64, 633–647. 10.1001/archpsyc.64.6.633 17548746

[B39] Keefe R. S. Fenton W. S. (2007). How should DSM-V criteria for schizophrenia include cognitive impairment? Schizophr. Bull. 33, 912–920. 10.1093/schbul/sbm046 17567627 PMC2632322

[B40] Kessels R. P. van Zandvoort M. J. Postma A. Kappelle L. J. de Haan E. H. (2000). The corsi block-tapping task: standardization and normative data. Appl. Neuropsychol. 7, 252–258. 10.1207/S15324826AN0704_8 11296689

[B41] Khan A. Yavorsky W. C. Liechti S. DiClemente G. Rothman B. Opler M.. (2013). Assessing the sources of unreliability (rater, subject, time-point) in a failed clinical trial using items of the positive and negative syndrome scale (PANSS). J. Clin. Psychopharmacol. 33, 109–117. 10.1097/JCP.0b013e3182776ebe 23277234

[B42] Landgraf S. Krebs M. O. Olié J. P. Committeri G. van der Meer E. Berthoz A.. (2010). Real world referencing and schizophrenia: are we experiencing the same reality? Neuropsychologia 48, 2922–2930. 10.1016/j.neuropsychologia.2010.05.034 20540956

[B43] Leeson V. C. Barnes T. R. Hutton S. B. Ron M. A. Joyce E. M. (2009). IQ as a predictor of functional outcome in schizophrenia: a longitudinal, four-year study of first-episode psychosis. Schizophr. Res. 107, 55–60. 10.1016/j.schres.2008.08.014 18793828 PMC2631642

[B44] Lewis S. Lieberman J. (2008). CATIE and CUtLASS: can we handle the truth? Br. J. Psychiatry 192, 161–163. 10.1192/bjp.bp.107.037218 18310570

[B45] Lezak M. D. Howieson D. B. Bigler E. D. Tranel D. (2012). Neuropsychological Assessment. NewYork, NY: Oxford University Press.

[B46] Loh S. Lamond N. Dorrian J. Roach G. Dawson D. (2004). The validity of psychomotor vigilance tasks of less than 10-minute duration. Behav. Res. Methods Instrum. Comput. 36, 339–346. 10.3758/BF03195580 15354700

[B47] Makara-Studziñska M. Wołyniak M. Partyka I. (2011). The quality of life in patients with schizophrenia in community mental health service-selected factors. J. Pre-Clin. Clin. Res. 5, 31–34. Available online at: http://jpccr.eu/fulltxt.php?ICID=978221

[B48] Malla A. Payne J. (2005). First-episode psychosis: psychopathology, quality of life, and functional outcome. Schizophr. Bull. 31, 650–671. 10.1093/schbul/sbi031 16006593

[B49] Marková H. Laczó J. Andel R. Hort J. Vlček K. (2015). Perspective taking abilities in amnestic mild cognitive impairment and Alzheimer's disease. Behav. Brain Res. 281, 229–238. 10.1016/j.bbr.2014.12.033 25541035

[B50] Mas-Expósito L. Amador-Campos J. A. Gómez-Benito J. Lalucat-Jo L. (2011). The World Health Organization quality of life scale brief version: a validation study in patients with schizophrenia. Qual. Life Res. 20, 1079–1089. 10.1007/s11136-011-9847-1 21290191

[B51] Matsui M. Sumiyoshi T. Arai H. Higuchi Y. Kurachi M. (2008). Cognitive functioning related to quality of life in schizophrenia. Prog. Neuropsychopharmacol. Biol. Psychiatry 32, 280–287. 10.1016/j.pnpbp.2007.08.019 17884266

[B52] Meltzer H. Y. McGurk S. R. (1999). The effects of clozapine, risperidone, and olanzapine on cognitive function in schizophrenia. Schizophr. Bull. 25, 233–255. 10.1093/oxfordjournals.schbul.a033376 10416729

[B53] Mesholam-Gately R. I. Giuliano A. J. Goff K. P. Faraone S. V. Seidman L. J. (2009). Neurocognition in first-episode schizophrenia: a meta-analytic review. Neuropsychology 23, 315–336. 10.1037/a0014708 19413446

[B54] Milev P. Ho B. C. Arndt S. Andreasen N. C. (2005). Predictive values of neurocognition and negative symptoms on functional outcome in schizophrenia: a longitudinal first-episode study with 7-year follow-up. Am. J. Psychiatry 162, 495–506. 10.1176/appi.ajp.162.3.495 15741466

[B55] Money J. Walker H. T. Alexander D. (1965). A Standardized Road-map Test of Direction Sense. Baltimore, MD: Johns Hopkins Press.

[B56] Narvaez J. M. Twamley E. W. McKibbin C. L. Heaton R. K. Patterson T. L. (2008). Subjective and objective quality of life in schizophrenia. Schizophr. Res. 98, 201–208. 10.1016/j.schres.2007.09.001 17919890 PMC2222889

[B57] Nuechterlein K. H. Green M. F. Kern R. S. Baade L. E. Barch D. M. Cohen J. D.. (2008). The MATRICS consensus cognitive battery, part 1: test selection, reliability, and validity. Am. J. Psychiatry 165, 203–213. 10.1176/appi.ajp.2007.07010042 18172019

[B58] O'Leary D. S. Flaum M. Kesler M. L. Flashman L. A. Arndt S. Andreasen N. C. (2000). Cognitive correlates of the negative, disorganized, and psychotic symptom dimensions of schizophrenia. J. Neuropsychiatry Clin. Neurosci. 12, 4–15. 10.1176/jnp.12.1.4 10678506

[B59] Osterrieth P. A. (1944). Le test de copie d'une figure complex: contribution a l'étude de la perception et de la memoir. Arch. Psychol. 30, 286–356.

[B60] Paradis M. (2008). Bilingualism and neuropsychiatric disorders. J. Neurolinguistics 21, 199–230. 10.1016/j.jneuroling.2007.09.002

[B61] PEBL (2012). The Psychology Experiment Building Language. Version 0.12, Test Battery 0.7. Michigan Technological University.

[B62] Peuskens J. Demily C. Thibaut F. (2005). Treatment of cognitive dysfunction in schizophrenia. Clin. Ther. 27(Suppl. A), S25–S37. 10.1016/j.clinthera.2005.07.015 16198199

[B63] Peuskens J. Gorwood P. (2012). How are we assessing functioning in schizophrenia? A need for a consensus approach. Eur. Psychiatry 27, 391–395. 10.1016/j.eurpsy.2011.02.013 21632218

[B64] Piskulic D. Olver J. S. Norman T. R. Maruff P. (2007). Behavioural studies of spatial working memory dysfunction in schizophrenia: a quantitative literature review. Psychiatry Res. 150, 111–121. 10.1016/j.psychres.2006.03.018 17292970

[B65] Preiss M. Bartoš A. Čermáková R. Nondek M. Benešová M. Rodriguez M. (2012). Neuropsychologická Baterie Psychiatrického Centra Praha. Praha: Psychiatrické centrum Praha.

[B66] Preiss M. Preiss J. (2006). Test Cesty. Bratislava, MD: Psychodiagnostika.

[B67] Prouteau A. Verdoux H. Briand C. Lesage A. Lalonde P. Nicole L.. (2005). Cognitive predictors of psychosocial functioning outcome in schizophrenia: a follow-up study of subjects participating in a rehabilitation program. Schizophr. Res. 77, 343–353. 10.1016/j.schres.2005.03.001 16085207

[B68] Rajji T. K. Mulsant B. H. (2008). Nature and course of cognitive function in late-life schizophrenia: a systematic review. Schizophr. Res. 102, 122–140. 10.1016/j.schres.2008.03.015 18468868

[B69] Reed R. A. Harrow M. Herbener E. S. Martin E. M. (2002). Executive function in schizophrenia: is it linked to psychosis and poor life functioning? J. Nerv. Ment. Dis. 190, 725–732. 10.1097/00005053-200211000-00001 12436011

[B70] Reitan R. M. Wolfson D. (1985). The Halstead-Reitan Neuropsychological Test Battery: Theory and Clinical Interpretation. Tuscon, AZ: Neuropsychology Press.

[B71] Rey A. (1964). L'examen Clinique en Psychologie. Paris: Presses Universitaries de France.

[B72] Ritsner M. S. (2007). Predicting quality of life impairment in chronic schizophrenia from cognitive variables. Qual. Life Res. 16, 929–937. 10.1007/s11136-007-9195-3 17404898

[B73] Rocca P. Giugiario M. Montemagni C. Rigazzi C. Rocca G. Bogetto F. (2009). Quality of life and psychopathology during the course of schizophrenia. Compr. Psychiatry 50, 542–548. 10.1016/j.comppsych.2008.12.002 19840592

[B74] Rodriguez M. M. V. (2012). Feasibility of Non-Pharmacological Intervention in Therapy of Cognition Deficit in Czech Schizophrenia Patients - Computer-assisted Cognitive Remediation. Prague: Facutly of Arts Charles University.

[B75] Rossi A. Mancini F. Stratta P. Mattei P. Gismondi R. Pozzi F.. (1997). Risperidone, negative symptoms and cognitive deficit in schizophrenia: an open study. Acta Psychiatr. Scand. 95, 40–43. 10.1111/j.1600-0447.1997.tb00371.x 9051159

[B76] Roy-Byrne P. Dagadakis C. Unutzer J. Ries R. (1996). Evidence for limited validity of the revised global assessment of functioning scale. Psychiatr. Serv. 47, 864–866. 10.1176/ps.47.8.864 8837160

[B77] Schultz K. (1991). The Contribution of solution strategy to spatial performance. Can. J. Psychol. 45, 474–491. 10.1037/h0084301

[B78] Spohn H. E. Strauss M. E. (1989). Relation of neuroleptic and anticholinergic medication to cognitive functions in schizophrenia. J. Abnorm. Psychol. 98, 367–380. 10.1037/0021-843X.98.4.367 2574202

[B79] Stirling J. White C. Lewis S. Hopkins R. Tantam D. Huddy A.. (2003). Neurocognitive function and outcome in first-episode schizophrenia: a 10-year follow-up of an epidemiological cohort. Schizophr. Res. 65, 75–86. 10.1016/S0920-9964(03)00014-8 14630300

[B80] Stuve T. A. Friedman L. Jesberger J. A. Gilmore G. C. Strauss M. E. Meltzer H. Y. (1997). The relationship between smooth pursuit performance, motion perception and sustained visual attention in patients with schizophrenia and normal controls. Psychol. Med. 27, 143–152. 10.1017/S0033291796004230 9122294

[B81] Tzeng D. S. Lung F. W. Chang Y. Y. (2004). Comparison of quality of life for people with schizophrenia and mental health of caregivers beteen commn-based and hospital-based services. Kaohsiung. J. Med. Sci. 20, 443–451. 10.1016/S1607-551X(09)70183-4 15506557 PMC11917851

[B82] Vargas M. L. Sanz J. C. Marín J. J. (2009). Behavioral assessment of the dysexecutive syndrome battery (BADS) in schizophrenia: a pilot study in the Spanish population. Cogn. Behav. Neurol. 22, 95–100. 10.1097/WNN.0b013e318192cd08 19506425

[B83] Ventura J. Hellemann G. S. Thames A. D. Koellner V. Nuechterlein K. H. (2009). Symptoms as mediators of the relationship between neurocognition and functional outcome in schizophrenia: a meta-analysis. Schizophr. Res. 113, 189–199. 10.1016/j.schres.2009.03.035 19628375 PMC2825750

[B84] Wechsler D. (1997). Manual for the Wechsler Memory Scale-III. San Antonio, TX: Psychological Corporation.

[B85] Weniger G. Irle E. (2008). Allocentric memory impaired and egocentric memory intact as assessed by virtual reality in recent-onset schizophrenia. Schizophr. Res. 101, 201–209. 10.1016/j.schres.2008.01.011 18276116

[B86] WHO group (1998). Development of the World Health Organization WHOQOL-BREF quality of life assessment. The WHOQOL Group. Psychol. Med. 28, 551–558. 10.1017/S0033291798006667 9626712

[B87] Wilson B. A. Alderman N. Burgess P. W. Emslie H. Evans J. J. (1996). Behavioural Assessment of the Dysexecutive Syndrome. London: Thames Valley Test Company.

[B88] Woods S. W. (2003). Chlorpromazine equivalent doses for the newer atypical antipsychotics. J. Clin. Psychiatry 64, 663–667. 10.4088/JCP.v64n0607 12823080

